# Increased frequency of systemic pro-inflammatory Vδ1^+^ γδ T cells in HIV elite controllers correlates with gut viral load

**DOI:** 10.1038/s41598-018-34576-4

**Published:** 2018-11-07

**Authors:** Gregory S. Olson, Sarah W. Moore, James M. Richter, John J. Garber, Brittany A. Bowman, Crystal A. Rawlings, Meaghan Flagg, Björn Corleis, Douglas S. Kwon

**Affiliations:** 1 The Ragon Institute of MGH, MIT and Harvard, Cambridge, Massachusetts, United States of America; 2 Division of Gastroenterology, Massachusetts General Hospital, Boston, Massachusetts, United States of America

## Abstract

γδ T cells predominate in the intestinal mucosa and help maintain gut homeostasis and mucosal immunity. Although HIV infection significantly alters these cells, what drives these perturbations is unclear. Growing evidence suggests that impaired intestinal immune function in HIV leads to chronic immune activation and disease progression. This occurs even in HIV controllers – individuals with undetectable HIV viremia without antiretroviral therapy (ART). We show that Vδ1^+^ cells, a subset of γδ T cells described as being important in intestinal barrier function, increase in frequency in HIV-infected individuals, including HIV controllers. These cells resemble terminally differentiated effector memory cells, producing the pro-inflammatory cytokines IFNγ, TNFα, and MIP-1β upon stimulation. Importantly, pro-inflammatory Vδ1^+^ cell frequency correlates with levels of HIV RNA in intestinal tissue but not in plasma. This study supports a model in which local viral replication in the gut in HIV controllers disrupts the phenotype and function of Vδ1^+^ cells, a cell type involved in the maintenance of epithelial barrier integrity, and may thereby contribute to systemic immune activation and HIV disease progression.

## Introduction

A small proportion of individuals infected with human immunodeficiency virus type 1 (HIV-1, hereafter HIV) maintain low or undetectable viremia in the absence of antiretroviral therapy (ART). Despite this, these so-called “HIV controllers” still demonstrate increased morbidity and mortality associated with chronic systemic inflammation^1–5^. In addition, they have detectable viral replication in the gut and impaired gut barrier function^6^. Studies of HIV controllers therefore provide an opportunity to explore the impact of HIV on intestinal immune function in the absence of the confounding effects of ART.

Current models of HIV disease progression suggest that HIV-associated disruption of the gastrointestinal tract results in microbial translocation across a compromised intestinal epithelial barrier and subsequent chronic immune activation, disease progression, and increased mortality in HIV disease^7,8^. However, the cell types involved with the compromised intestinal barrier and subsequent chronic inflammation are not well understood. Gamma delta (γδ) T cells are an ‘innate’ T cell type that expresses a semi-invariant T cell receptor (TCR). The differential usage of the Vδ1 or Vδ2 genes in the rearranged TCR differentiate two main subsets of human γδ T cells^9^. The recognition of both microbial products and stressed host cells allows γδ T cells to play an important role in immune responses against infections in general and viruses in particular^10–12^. While Vδ2^+^ cells primarily circulate in blood, Vδ1^+^ cells primarily localize within the mucosa of the gut as intraepithelial lymphocytes (IELs) and help to maintain epithelial function^11^. Their connection to HIV-associated gut dysfunction remains incompletely characterized.

Progressive HIV infection drastically changes peripheral γδ T cell subsets^13–19^, including a depletion of Vδ2^+^ cells and an expansion of Vδ1^+^ cells in circulating blood^16–18^. Controlling viremia with ART does not fully correct the inversion of the normal ratio of peripheral γδ T cell subsets^16,17^. The expanded Vδ1^+^ cells also behave differently, becoming more likely to produce the pro-inflammatory cytokines IFNγ, TNFα^13,19^, IL-17A^14^, and MIP1β^15,20^. Whether Vδ1^+^ cells are disturbed in HIV controllers is currently unknown.

To better understand HIV-associated alterations in Vδ1^+^ populations and their potential role in gut dysfunction, we characterized Vδ1^+^ cell phenotype and function in HIV-infected individuals, including HIV controllers. Since local viral replication in the gut has been implicated in the disruption of resident immune subsets and the impairment of intestinal barrier integrity^21,22^, we hypothesized that Vδ1^+^ cells in HIV controllers would resemble those in chronic progressive HIV infection, and that the alterations in Vδ1^+^ cell frequency and phenotype would be associated with local viral replication within intestinal tissue and not with replication in the blood.

## Results

### Increased frequency of peripheral Vδ1^+^ cells in HIV controllers

**Figure 1:**
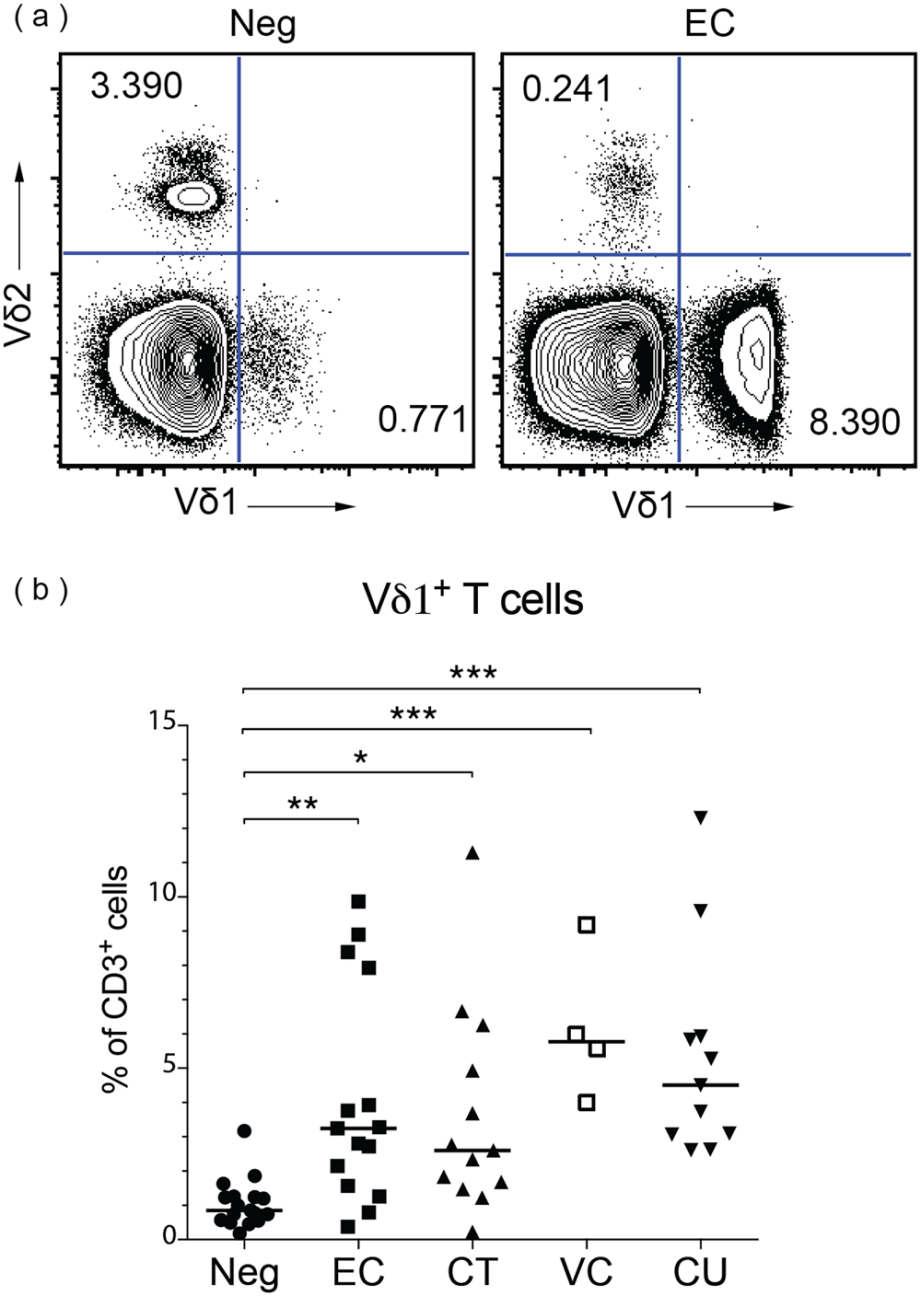
Vδ1^+^ cells expanded in HIV-infected white subjects, despite controlled viremia. PBMCs were assessed by flow cytometry and viable CD3^+^ cells were analyzed for expression of Vδ1 and Vδ2. (**a**) Representative flow plots from an HIV- uninfected subject (Neg) and an elite controller (EC) are shown. The numbers in the quadrants represent the percentage of total CD3^+^ cells. (**b**) Summary data showing the median percentage of viable CD3^+^ cells that are Vδ1^+^ for Neg (n = 17), EC (n = 15), chronic treated (CT; n = 13), viremic controller (VC; n = 4), and chronic untreated (CU; n = 11) subjects. The medians of the cohorts were significantly different (p < 0.0001) as assessed by the Kruskal-Wallis test. Dunn’s multiple comparison tests were used to assess differences between Neg and each HIV- infected group. *p < 0.05; **p < 0.01; ***p < 0.001.

Because the Vδ1^+^ cell subset is incompletely characterized in HIV controllers, we first used flow cytometry to analyze Vδ1^+^ cell subsets in PBMCs from HIV-uninfected control subjects and HIV-infected subjects from the following cohorts: HIV controllers (further subdivided into elite controllers (EC; HIV viral load (VL) undetectable) and viremic controllers (VC; HIV VL <2000 copies/ml)), ART treated, and ART untreated individuals (Table 1). These cells were defined as CD3^+^ Vδ1^+^ Vδ2^−^ (Fig. 1a and see Supplementary Fig. S9). Although Vδ2^+^ cells represent the majority of circulating γδ T cells in healthy white individuals^9,11,23,24^, the ratio of Vδ2^+^ to Vδ1^+^ cells in healthy individuals is inverted among some self-reported racial groups^25,26^. Initial analyses were therefore conducted on subsets defined by self-reported race.

**Table 1:** Clinical characteristics of white subjects.

	HIV Negative	Elite Controller	Chronic Treated	Viremic Controller	Chronic Untreated
# subjects	17	15	13	4	11
Age (years)	43 ± 11	49 ± 9	45 ± 5	53 ± 8	40 ± 8
Male (%)	53	93	69	100	64
Hispanic/Latino (#)	1	0	0	0	2
CD4 + T Cells (/μL)		948 ± 228	679 ± 359	733 ± 172	464 ± 169
Plasma VL (copies/mL)		undetectable	undetectable	163 ± 53	44,559 ± 36,903
Days since diagnosis		6,386 ± 3,845	4,873 ± 2,549	8,133 ± 891	5,371 ± 2,441
HCV (#)^a^	0	2	4	0	2

Values for age, CD4^+^ T cell count, and days since diagnosis represent mean ± standard deviation. ^a^HCV (#) represents the number of subjects co-infected with Hepatitis C virus. CD4^+^ T cell count and plasma VL not determined for HIV negative cohort.

We found that the frequency of Vδ1^+^ cells among total T cells significantly increased in the blood of all white HIV-infected subjects compared to uninfected controls (Fig. 1). This increase was seen even in HIV-infected subjects with undetectable viremia. The Vδ1^+^ cell frequencies in EC (median 3.25%, range 0.38–9.87%), CT subjects (median 2.60%, range 0.22–11.3%), VC (median 5.78%, range 4.00–9.18%), and CU subjects (median 4.51%, range 2.61–12.3%) were all significantly higher than that in uninfected controls (median 0.85%, range 0.18–3.17%).

Interestingly, African American subjects showed a different pattern, with an increased Vδ1^+^ cell frequency only observed within the CU group (see Supplementary Fig. S1 and Supplementary Table 1). Due to this observation, the known variations in γδ cell subsets across self-reported racial groups^25,26^, and limited access to intestinal samples from other self-reported racial groups, further phenotypic analysis of peripheral Vδ1^+^ cells was only performed in white subjects.

To confirm that the differences in gender composition in our cohorts did not explain the differences in Vδ1^+^ cell frequency observed, we repeated the analysis after excluding female subjects. Because the Vδ1^+^ cell frequencies in male EC remained significantly increased compared to male uninfected controls (p < 0.05 and see Supplementary Fig. S2), we did not segment by gender in analyses.

Previous reports have shown that the increased frequency of Vδ1^+^ cells in HIV infection reflects increased absolute counts as well^16–18^. We used clinical CD4^+^ T cell counts to approximate absolute numbers of Vδ1^+^ cells in our HIV-infected cohorts. There was a significant correlation between the Vδ1^+^ cell frequency of CD3^+^ cells and the absolute count (R^2^ = 0.6536, p < 0.0001). In addition, the patterns of Vδ1^+^ cells in EC and other HIV-infected cohorts remained unchanged (see Supplementary Figure S3), suggesting that the increased frequencies of Vδ1^+^ cells during HIV infection represent an expansion in absolute numbers of these cells.

### Peripheral Vδ1^+^ cells display an effector memory phenotype in HIV controllers

To further characterize the phenotype of the Vδ1^+^ cell population, expression of cell surface markers CD45RA and CD27 were used to distinguish four subsets of γδ T cells^27–29^, that correspond to memory subsets of αβ T cells^30^. Double positive cells (CD45RA^+^CD27^+^) demonstrate characteristics of naïve T cells, CD45RA^−^CD27^+^ cells resemble central memory cells, while CD45RA^−^CD27^−^ cells resemble effector memory cells. CD45RA^+^CD27^−^ γδ cells are the proposed equivalent of terminally differentiated effector memory cells^27,28^.

**Figure 2:**
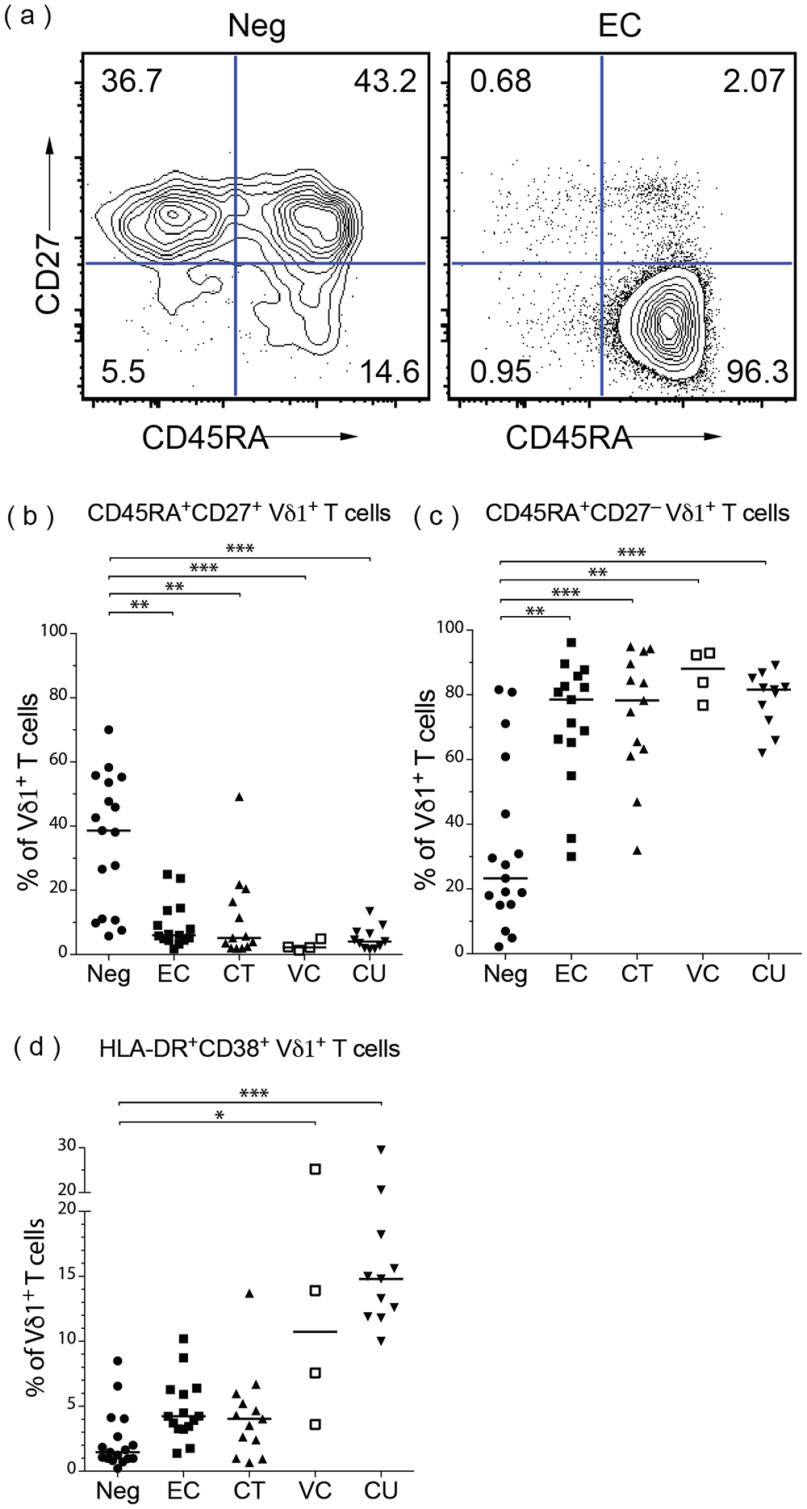
Vδ1^+^ cells in HIV controllers display an activated, effector-like phenotype. PBMCs were characterized by flow cytometry and the phenotypes of viable CD3^+^Vδ1^+^ cells were assessed. (**a**) Representative flow plots from an HIV-uninfected subject (Neg) and an elite controller (EC). The numbers in each quadrant represent the percentage of CD3^+^Vδ1^+^ cells. (**b**–**d**) Summary data of median percentages of Vδ1^+^ cells that are CD45RA^+^CD27^+^ (b), CD45RA^+^CD27^−^ (c), or HLA-DR^+^CD38^+^ (d) for Neg (n = 17), EC (n = 15), CT (n = 13), VC (n = 4), and CU (n = 11) subjects. The medians of the cohorts were significantly different for all three (b-d) summary graphs (p < 0.0001) as assessed by the Kruskal-Wallis test. Dunn’s multiple comparison tests were used to assess differences between HIV^−^ and HIV^+^ groups. *p < 0.05; **p < 0.01; ***p < 0.001.

We observed shifts in peripheral Vδ1^+^ cells away from a CD45RA^+^CD27^+^ naïve phenotype towards a CD45RA^+^CD27^−^ terminally differentiated phenotype in all HIV-infected cohorts, including those with controlled viremia (Fig. 2a–c). Specifically, the proportion of Vδ1^+^ cells displaying the CD45RA^+^CD27^+^ naïve phenotype was significantly reduced in EC (median 6.03%, range 1.73–25%) and VC (median 2.24%, range 1.31–4.89%) compared to uninfected controls (median 38.60%, range 5.73–70%) (Fig. 2a,b). Strikingly, almost all of the Vδ1^+^ cells displayed a terminally differentiated CD45RA^+^CD27^−^ phenotype in HIV-infected cohorts. The proportions of Vδ1^+^ cells that were CD45RA^+^CD27^−^ in both EC (median 78.50%, range 30.00–96.20%) and VC (median 88.05%, range 76.80–92.90%) were significantly higher than that in uninfected controls (median 23.30%, range 2.17–81.60%) (Fig. 2c).

We next assessed if Vδ1^+^ cells displayed additional evidence of activation. The percentage of Vδ1^+^ cells expressing both activation markers CD38 and HLA-DR^31–33^ increased significantly relative to uninfected controls only in cohorts with detectable viremia (Fig. 2d). Although cohorts with undetectable plasma VL trended towards increased median percentages of CD38^+^HLA-DR^+^ Vδ1^+^ cells, the increase did not reach statistical significance (Fig. 2d).

To determine if direct viral infection could explain the activation, we measured CD4 expression on Vδ1^+^ cells. Because the median frequency of CD4 on Vδ1^+^ cells was less than 10% in each cohort (see Supplemental Figure S4,), we did not pursue direct infection as a driver for the population level activation changes observed.

### Increased frequencies of peripheral Vδ1^+^ cells producing pro-inflammatory cytokines in HIV controllers

We also characterized whether these Vδ1^+^ cells had altered patterns of cytokine production in addition to increased markers of activation. Based on recent reports of γδ T cell function^13–15,19^, we measured the production of the pro-inflammatory cytokines IFNγ, TNFα, MIP-1β, and IL-17A by T cell subsets in response to various stimuli using intracellular cytokine staining. Because IL-17A production in γδ T cells was not detected after any condition—although it was in αβ T cells (see Supplementary Fig. S5)—we restricted subsequent analyses to IFNγ, TNFα, and MIP-1β.

**Figure 3:**
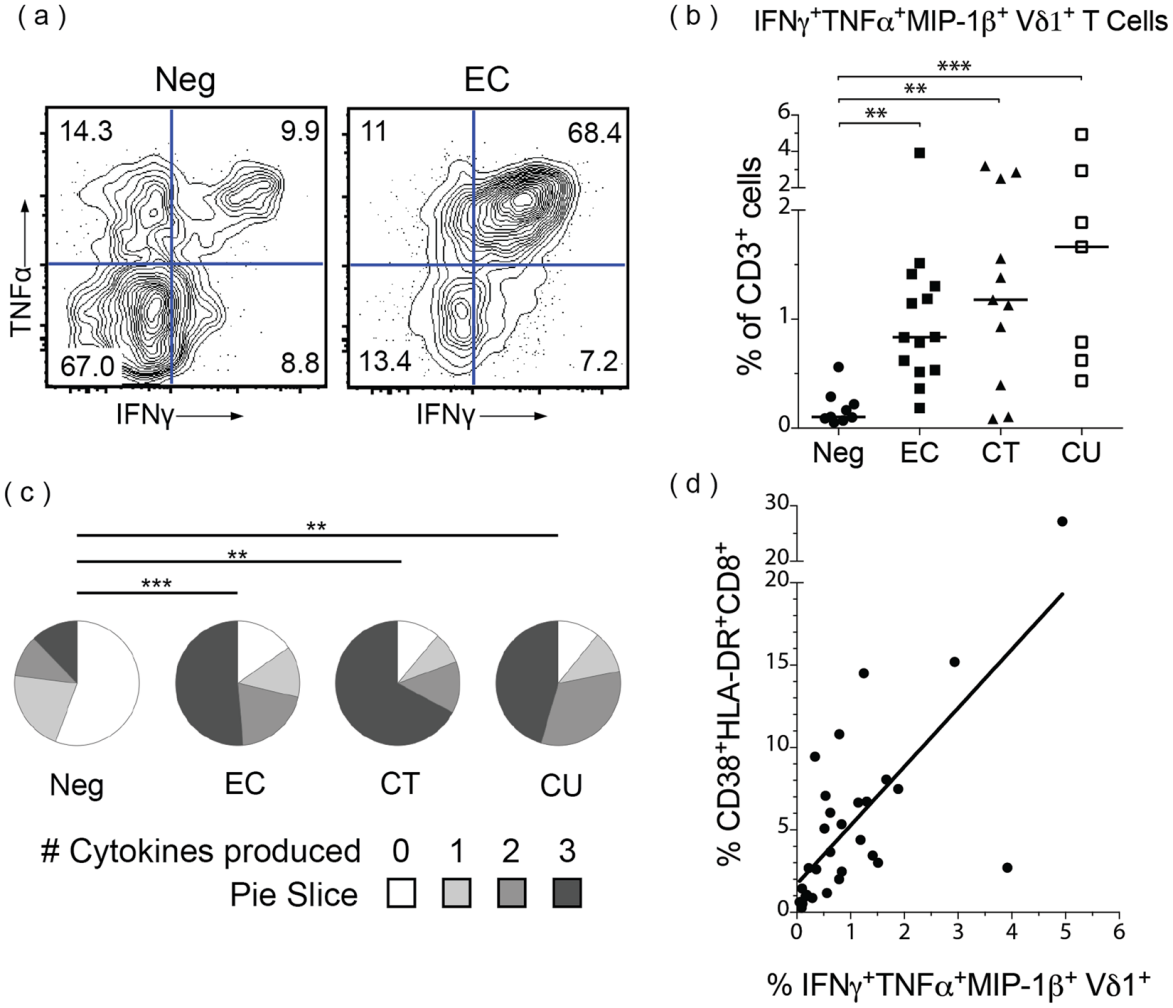
Vδ1^+^ cells in HIV controllers produce inflammatory cytokines. PBMCs were stimulated with PMA/ionomycin for 6 hours and cytokine production was measured by intracellular cytokine staining. Viable CD3^+^Vδ1^+^ cells were analyzed for production of IFNγ, TNFα, MIP1β, and IL-17A. (**a**) Representative flow plots from an HIV-uninfected subject (Neg) and an elite controller (EC) showing percentages of Vδ1^+^ cells expressing IFNγ and TNFα. (**b**) Summary data showing the median percentage of viable CD3^+^ cells that are IFNγ^+^TNFα^+^MIP1β^+^ Vδ1^+^ for Neg (n = 9), EC (n = 14), CT (n = 11), and CU (n = 7) subjects. The medians were significantly different (p = 0.0008) as assessed by the Kruskal-Wallis test. Dunn’s multiple comparison tests were used to assess differences between HIV^−^ and HIV^+^ groups. (**c**) The median distribution of Vδ1^+^ cells producing the indicated number of cytokines in each cohort. The distributions in HIV-infected cohorts were significantly different than that of HIV-uninfected subjects as assessed by the permutation analysis in SPICE (n = 10,000 iterations). *p < 0.05; **p < 0.01; ***p < 0.001. (**d**) Spearman’s rank correlation between the frequency of pro-inflammatory IFNγ^+^TNFα^+^MIP1β^+^ Vδ1^+^ cells of total CD3^+^ cells and the frequency of activated (HLA-DR^+^CD38^+^) CD8^+^ T cells out of total CD8^+^ T cells; Spearman’s ρ = 0.7107, p < 0.0001; the same subjects as in c, without CT (30 total subjects).

Vδ1^+^ cells from HIV-infected individuals produced significantly different cytokine signatures than those from uninfected controls upon stimulation with PMA/ionomycin, a potent non-specific activator of T cells. Frequencies of Vδ1^+^ cells producing IFNγ, TNFα, and MIP-1β (IFNγ^+^TNFα^+^MIP-1β^+^ Vδ1^+^ cells, hereafter “pro-inflammatory Vδ1^+^ cells”) were increased approximately ten-fold in HIV-infected individuals, even in the absence of detectable viremia (Fig. 3). Specifically, both EC (median 0.84% of CD3^+^ cells; range 0.19–3.92%) and CT subjects (median 1.18%; range 0.09–3.20%) had significantly higher frequencies of pro-inflammatory Vδ1^+^ cells than uninfected controls (median 0.10%; range 0.05–0.56%) (Fig. 3a,b).

Vδ1^+^ cells did not consistently produce any of the cytokines measured upon incubation with other stimuli. Stimulation with gag peptide pools resulted in no specific cytokine production in γδ T cells (see Supplementary Fig. S6), although responses were seen in CD8^+^ T cells in HIV-infected individuals (see Supplementary Fig. S7). Contrary to previous reports^14^, *C. albicans* stimulation did not lead to increased cytokine production in Vδ1^+^ cells (see Supplementary Fig. S6).

Having shown that pro-inflammatory Vδ1^+^ cells increased as a percentage of CD3^+^ cells in HIV infection (Fig. 3b), we investigated the proportion of cells within the Vδ1^+^ subset that produced pro-inflammatory cytokines. We found that a greater proportion of Vδ1^+^ cells from HIV-infected individuals produced the pro-inflammatory cytokines tested compared with uninfected controls (Fig. 3c). Although Vδ1^+^ cells not producing any cytokine upon PMA/ionomycin stimulation were the majority in uninfected controls (median 55.80%; range 20.23–74.09%), they were only a small fraction of total Vδ1^+^ cells in EC (median 15.45%; range 5.71–30.85%), CT subjects (median 11.37%; range 2.12–47.89%), and CU subjects (median 10.98%; range 2.79–30.56%) (Fig. 3c).

We next determined whether pro-inflammatory Vδ1^+^ cells might be associated with chronic immune activation observed during HIV infection^31^. We found the frequency of peripheral pro-inflammatory Vδ1^+^ cells significantly correlated with chronic immune activation as measured by the percentage of CD38^+^HLA-DR^+^ among Vδ1^−^Vδ2^−^CD8^+^ T cells) (Spearman’s ρ = 0.7107; p < 0.0001) (Fig. 3d). To avoid the confounding effects introduced by the interaction of ART and chronic immune activation, we excluded CT subjects from this analysis.

### Vδ1^+^ cells are present in the intestinal mucosa in HIV controllers

**Figure 4:**
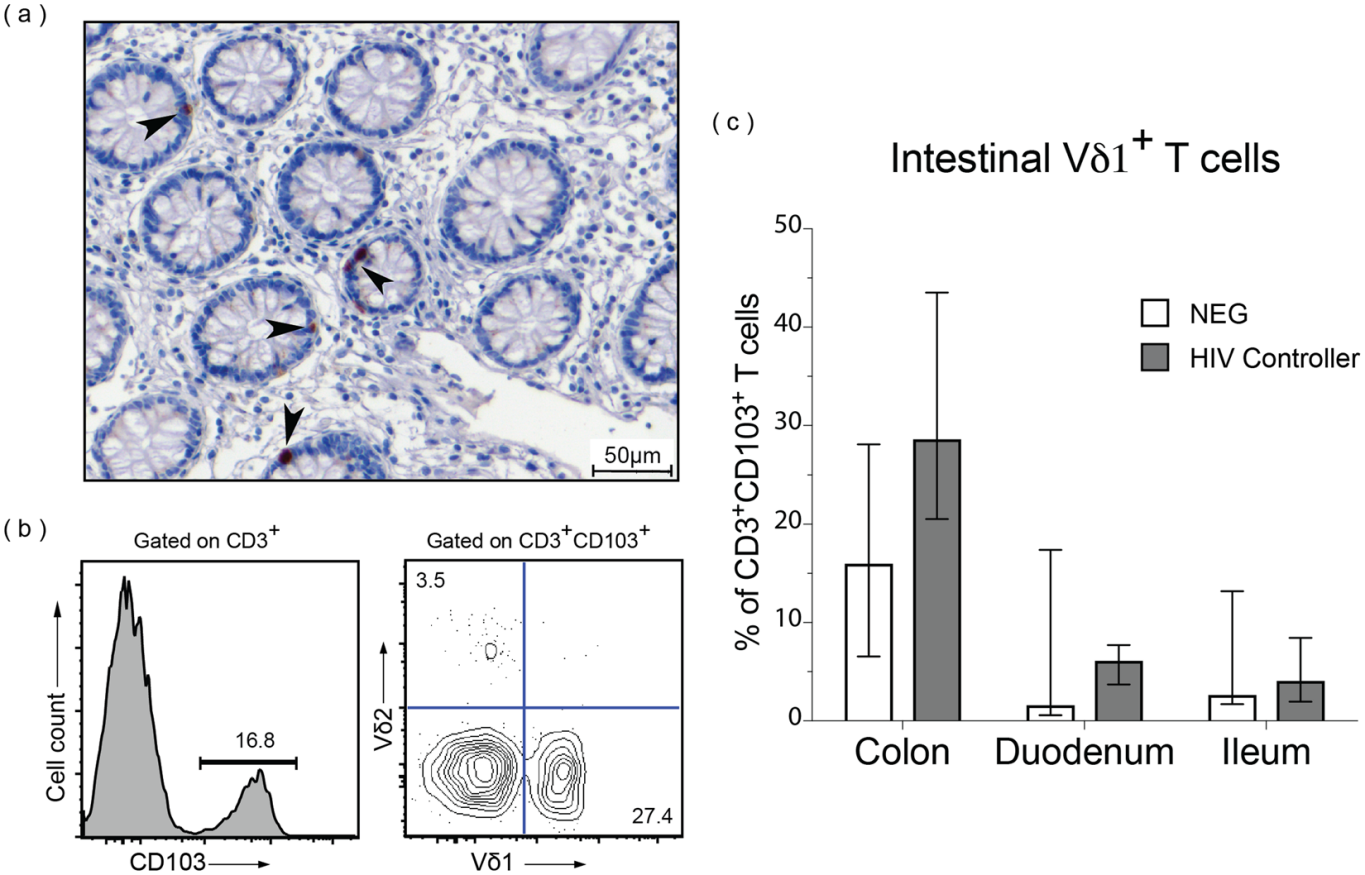
A large proportion of CD3^+^CD103^+^ mucosal-associated T cells in colon are Vδ1^+^. (**a**) A representative 20X image of a colon pinch biopsy stained with a monoclonal antibody against the γδ TCR. Note the intraepithelial location of the γδ^+^ cells (arrowheads). (**b**) Cells isolated from fresh intestinal pinch biopsies were analyzed by flow cytometry and viable CD3^+^ cells were gated for CD103 expression and subsequently analyzed for Vδ1 and Vδ2 expression. (**c**) Summary data showing the median percentage of CD3^+^CD103^+^ cells that are Vδ1^+^ in the transverse colon, duodenum, and terminal ileum of HIV-uninfected (Neg) subjects (n = 3) or HIV controllers (viremic controllers (n = 3), elite controllers (n = 3)).

Because phenotypic and functional perturbations of peripheral Vδ1^+^ cells are seen even in cohorts with undetectable viremia, we hypothesized that the perturbations might rather be associated with local viral replication in intestinal tissues where Vδ1^+^ cells reside. To confirm that γδ T cells localize in the intestinal mucosa of HIV controllers, we used IHC to determine the percentage of γδ cells within the colonic epithelial layer. We found that 84% (265/317) of γδ T cells were intraepithelial in the colonic mucosa of EC (Fig. 4a), similar to the distribution in uninfected controls (79%, 96/122 cells).

We further confirmed that γδ T cells reside in the gut mucosa of HIV controllers by flow cytometry of samples isolated from intestinal pinch biopsies. Total populations of mucosal-associated T cells were first identified as CD3^+^CD103^+^ cells^34^ and then the frequency of Vδ1^+^Vδ2^−^ cells was measured (Fig. 4b). Vδ1^+^ cells constituted a large percentage of intestinal CD3^+^CD103^+^ cells and predominated in the colon of viremic controllers (median 38.0%; range 27.1–60.10%), elite controllers (median 27.4%; range 0.83–30.0%) and uninfected controls (median 16.10%; range 6.55–28.10%) (Fig. 4c).

### Frequency of pro-inflammatory Vδ1^+^ correlate with relative gut VL in HIV controllers

We next addressed whether frequencies of peripheral pro-inflammatory Vδ1^+^ cells were related to systemic or local gut viral replication in HIV controllers. Relative gut viral load (VL) was quantified from pinch biopsies and Vδ1^+^ cell functional analysis was performed on 12 EC, 2 VC, and 3 uninfected controls. Similar to a previous report^6^, we were able to detect HIV viral RNA in multiple gut compartments of the majority of HIV controllers, despite undetectable viral RNA in PBMCs and an undetectable clinical plasma VL (see Supplementary Table S2).

**Figure 5:**
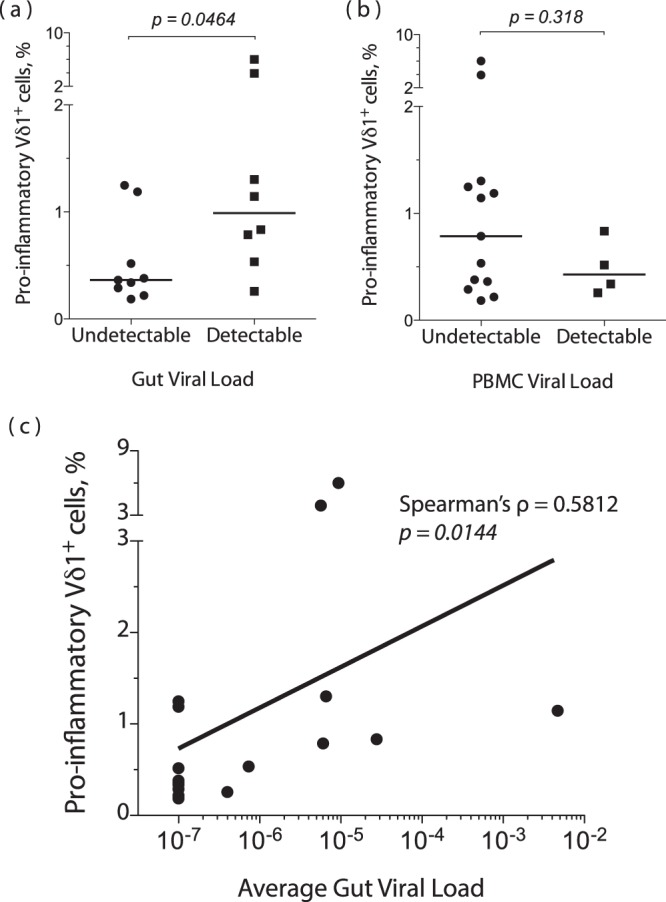
Levels of pro-inflammatory Vδ1^+^ cells correlate with gut-associated, but not plasma-associated, relative viral load. HIV *gag* RNA was quantified in PBMCs and intestinal pinch biopsies by qRT-PCR for HIV-uninfected (Neg) (n = 3), EC (n = 12), and VC (n = 2) subjects. Relative viral load (VL) was determined after normalizing the copies of *gag* to copies of *RPS9*. The average gut VL represents the mean VL across three intestinal compartments (transverse colon, duodenum, and terminal ileum). Subjects were divided into groups with detectable or undetectable viral load in any gut compartment or in the blood. (**a**,**b**) Summary data showing the percentage of pro-inflammatory (IFNγ^+^TNFα^+^MIP1β^+^) Vδ1^+^ cells out of total CD3^+^ cells, grouped by gut VL (**a**) or PBMC VL (**b**). Horizontal lines represent the median. A two-tailed Mann-Whitney test was used to compare the medians. (**c**) Spearman’s rank correlation between the average gut VL and the percentage of CD3^+^ cells that are pro-inflammatory Vδ1^+^.

Significantly more peripheral pro-inflammatory Vδ1^+^ cells were present in subjects with detectable viral RNA in any gut compartment than in those with no detectable virus (p = 0.0464) (Fig. 5a). To confirm that the expansion of pro-inflammatory Vδ1^+^ cells was not explained by peripheral viral burden, we repeated the dichotomization using detectable virus measured either by PBMC-associated viral RNA (measured in the same manner as gut-associated virus) or plasma viremia (measured in the clinical setting). No significant difference was found in the frequencies of peripheral pro-inflammatory Vδ1^+^ cells between individuals dichotomized by PBMC-associated viral RNA (Fig. 5b) or between HIV-infected individuals dichotomized by plasma VL (see Supplementary Figure S8). Furthermore, Spearman’s ranked correlation coefficient showed that the frequency of peripheral pro-inflammatory Vδ1^+^ cells significantly correlated with the average relative gut VL (Spearman’s ρ = 0.5812, p = 0.0144) (Fig. 5c). Thus, the frequency of peripheral pro-inflammatory Vδ1+ cells was closely associated with intestinal but not peripheral levels of HIV.

## Discussion

In this study, we found that peripheral Vδ1^+^ cells are increased in frequency in HIV controllers and produce multiple pro-inflammatory cytokines, similar to ART-treated and -untreated individuals infected with HIV. These perturbations are correlated with VL in the gut, but are independent of peripheral viral burden. We believe the increased frequency of Vδ1^+^ cells seen in this study represents a true expansion based on the phenotypic alterations and prior studies of chronically HIV-infected cohorts^16,17,35^. The expansion of Vδ1^+^ cells in EC and VC seen in this study indicates that alterations in Vδ1^+^ cells occur even with immunologic control of HIV. Interestingly, we did not observe an expansion of Vδ1^+^ cells in a cohort of African American ECs, emphasizing the need raised by previous reports to account for race in studies on γδ subsets^25,26^.

The origin of the expanded Vδ1^+^ population remains unknown and is a promising area of future investigation. Vδ1^+^ populations acting as IELs differ from those in the lamina propria in their ontogeny, TCR repertoire, and their propensity to circulate in vasculature at steady state^36,37^. Deeper characterization of changes in cell localization during HIV infection and the application of next generation TCR sequencing of Vδ1^+^ clones in the gut and blood of HIV-infected individuals would help differentiate the possible source of the expanded Vδ1^+^ population in our cohorts.

Direct infection of γδ cells has been reported in the literature^38,39^, but the rates of infection in these cells are very low and unlikely to fully account for the drastic phenotypic changes seen in the majority of Vδ1^+^ cells. Although a direct assessment of HIV infection was not conducted in this study, few Vδ1^+^ cells from each cohort expressed CD4 (see Supplemental Figure S4). Interestingly, HIV-infected individuals did have significantly decreased percentages of Vδ1^+^ cells that expressed CD4. Future studies to explore the possibility of depletion of CD4^+^ Vδ1^+^cells by direct infection might add to the growing appreciation of γδ cells as HIV targets^38^.

The observed shift of peripheral Vδ1^+^ cells towards a pro-inflammatory Th1 profile in HIV-infected individuals is consistent with prior findings^13,14,19^, but has never, to our knowledge, been shown in a cohort with viral suppression in the absence of ART. Contrary to previous reports^14,40^, we did not detect specific IL-17A expression in peripheral γδ T cells stimulated with *C. albicans* or PMA/ionomycin. This might be explained by the different approaches; our study assayed *ex vivo* cytokine production using short incubation times (6 hours), rather than following an extended (>1 week) *in vitro* expansion phase.

Chronic inflammation has been linked to disease progression and increased morbidity in HIV infection in general^41,42^ and in ECs in particular^1,4,5^. A disrupted intestinal epithelial barrier is thought to lead to microbial translocation from the gut into the periphery and subsequent immune activation, thereby exacerbating the chronic inflammation in HIV infection^8^. Multiple reports support large perturbations of Vδ1^+^ cell populations at mucosal surfaces, although the specifics of these changes differ: some groups report they expand in the rectum^35^ and duodenum^43,44^ of HIV-infected individuals, while others report they decrease in the duodenum^29^ and the vaginal mucosa^45^ during infection. These changes of Vδ1^+^ cells in the intestinal mucosa, their ability to robustly respond to stressed epithelial cells, and their role in maintaining intestinal barrier integrity support their possible involvement in the proposed immune activation that drives disease progression^11,24,44^. In fact, a study in rhesus macaques linked levels of microbial translocation in Simian Immunodeficiency Virus (SIV) infection with the expansion of Vδ1^+^ cells^39^. Another report connected microbial translocation, Vδ1^+^ cell expansion, and disease progression in the setting of acute HIV infection^28^. Our data add an additional component to support a model in which Vδ1^+^ cells might be involved in chronic inflammation in HIV resulting from loss of the intestinal barrier integrity. Specifically, we propose that local viral replication in intestinal tissues rather than systemic viral burden leads to the increased frequency, activated phenotype, and pro-inflammatory function of Vδ1^+^ cells in HIV-infected individuals. While a perturbed Vδ1^+^ subset might contribute to impaired gut function and chronic inflammation in EC and other HIV-infected subjects, further work is needed to explore the origins and mechanistic role of these cells in this process. Investigating this link will yield insight into the mechanisms of HIV-induced impairment of the intestinal immune system and potentially lead to novel interventions to decrease HIV-related chronic inflammation by targeting Vδ1^+^ cells.

## Methods

### Study participants

The study was approved by the Massachusetts General Hospital Institutional Review Board and was performed in accordance with the approved guidelines. All participants provided written informed consent. HIV elite controllers (EC) were defined by ≥3 undetectable plasma HIV-1 RNA (plasma VL) measurements spanning ≥12 months without ART. Chronic treated (CT) subjects were defined by undetectable plasma VL measurements while on ART for ≥12 months prior to sample date. Viral blips <200 copies/mL were not exclusion criteria in either of these groups if the plasma VL became undetectable within a year. HIV viremic controllers (VC) were defined by detectable low levels of viremia, plasma VL of <2000 copies/mL, for ≥12 months without ART. Chronic untreated (CU) subjects had plasma VL >2000 copies/mL for ≥12 months without ART. Subject characteristics, including ethnicity and sex, were self-reported on intake forms.

### Flow cytometry analysis

Peripheral blood mononuclear cells (PBMCs) from venous blood collected in acid citrate dextrose tubes were separated by centrifugation on a Histopaque gradient and cryopreserved in liquid nitrogen. Cryopreserved PBMCs were thawed and 5 × 10^6^ cells were stained for viability with LIVE/DEAD Fixable Violet Dead Cell Stain Kit (Life Technologies). Surface markers were identified with the following mouse monoclonal antibodies (mAbs): FITC anti-Vδ1 (clone TS8.2, Thermo Fisher Scientific), PE anti-Vδ2 (B6, BD Biosciences), PE-CF594 anti-CD3 (UCHT1, BD Biosciences), Brilliant Violet 605 (BV605) anti-CD4 (RPA-T4, BD Biosciences), v500 anti-CD8 (SK1, BD Biosciences), APC-H7 anti-CD27 (M-T271, BD Biosciences), PE-Cy5 anti-CD45RA (HI100, BD Biosciences), Alexa Fluor 700 (AF700) anti-HLA-DR (G46-6, BD Biosciences), AF647 anti-CD38 (HIT2, Biolegend), PE-Cy7 anti-CD103 (Ber-ACT8, Biolegend), V450 anti-CD19 (HIB19, BD Biosciences), and Pacific Blue anti-CD14 (M5E2, BD Biosciences). Stained cells were fixed with 2% paraformaldehyde before running on an LSRII flow cytometer (BD Biosciences). Flow data were analyzed with FlowJo (TreeStar).

### Intracellular cytokine staining analysis

Cryopreserved PBMCs were thawed and rested overnight at 37 °C, 5% CO_2_ at 2 × 10^6^ cells/mL of R^+^ media (RPMI-1640 Medium (Sigma-Aldrich) supplemented with 10 mM HEPES buffer, 2mM L-glutamine, 50 IU/mL Penicillin, 50 μg/mL Streptomycin) with 10% (v/v) FBS (Sigma-Aldrich). Cells were resuspended at 4 × 10^6^/mL in R^+^ with 10% FBS, GolgiPlug (1.0 μg/mL, BD Biosciences), and soluble anti-CD28/CD49d mAbs (BD Biosciences). Stimuli included media only, a pool of gag overlapping peptides (2 μg/mL), *C. albicans* (10^6^ bodies/mL), or phorbol 12-myristate 13-acetate (PMA; 50 ng/mL) with ionomycin (1 μg/mL) (PMA/ionomycin, Ebioscience). After 6 hours at 37 °C, cells were stained with LIVE/DEAD Fixable Violet Dead Cell Stain Kit (Life Technologies) before intracellular cytokine staining with the Cytofix/Cytoperm Kit (BD Biosciences) according to the manufacturer’s instructions.

Cells were stained prior to permeabilization with the following mouse mAbs: FITC anti-Vδ1 (clone TS8.2, Thermo Fisher Scientific), PerCP anti-Vδ2 (B6, Biolegend), BV785 anti-PD-1 (EH12.2H7, Biolegend), BV510 anti-CD3 (UCHT1, BD Biosciences), BV605 anti-CD4 (RPA-T4, BD Biosciences), and APC-H7 anti-CD8 (SK1, BD Biosciences).

Intracellular antigens were detected using mouse mAbs: AF647 anti-IL-17A (BL168, Biolegend), AF700 anti-TNFα (mAb11, BD Biosciences), PE anti-MIP-1β (D21-1351, BD Biosciences), and PE-Cy7 anti-IFNγ (B27, BD Biosciences).

Data acquisition was performed with an LSRII flow cytometer (BD Biosciences) and analyzed with FlowJo software (TreeStar). No background subtraction was performed on cytokine-producing subsets. Cytokine expression analysis was performed using SPICE version 5.1, from http://exon.niaid.nih.gov^46^.

### Candida albicans preparation

*Candida* was prepared as previous reported^14^. Briefly, *C. albicans* (Ca) was grown in RPMI 1640 medium for 2 days, washed twice in PBS, autoclaved, and used at a final concentration of 10^6^ bodies/mL.

### Mononuclear cell isolation from intestinal tissue

Ten intestinal pinch biopsies from each site were collected by colonoscopy (transverse colon and terminal ileum) and upper endoscopy (duodenum) and placed immediately into 4 °C gut-wash (R^+^ supplemented with 222 μg/mL piperacillin, 28 μg/mL tazobactam, and 2.5 μg/mL amphotericin B) and kept on ice <2 hours until processing by an adapted collagenase type II protocol^47^. Briefly, tissue was mechanically disrupted by passage through a 16-gauge needle before a 20 minute incubation at 37 °C with 0.50 mg/mL collagenase type II (*Clostridium histolyticum*, Sigma-Aldrich). The cells in the supernatant were filtered through a 70 μm cell strainer and resuspended in gut-wash with 10% FBS. This process was repeated on the remaining tissue and the combined cells from both rounds were kept on ice until further analysis.

### Immunohistochemical (IHC) staining and quantification of γδ cells in intestinal tissue

Serial sections (4 μm) of formalin-fixed paraffin-embedded pinch biopsies were stained for the TCR γ chain by an adapted protocol^48^. Briefly, epitope retrieval in 10 mM sodium citrate buffer (pH6.5) for 2 minutes in a Decloaking Chamber (Biocare Medical) preceded staining with anti-TCRγ mouse mAb (clone γ3.20, Thermo Scientific) and the DAB Envision^+^ system (Dako). Identification of DAB^+^ cells in 20X images (TissueFAXS scanning system; Tissuegnostics) was assisted by HistoQUEST (Tissuegnostics) image analysis software and the percentage of intraepithelial γδ T cells was quantified for 2 slides per biopsy.

### Detecting and quantifying HIV viral load by qRT-PCR

Pinch biopsies of intestinal tissue were mechanically homogenized using a roto-stator (VWR) and a QIAshredder column. RNA was extracted using the RNeasy kit (Qiagen). Quantitative reverse transcription-PCR (qRT-PCR) was performed on a Roche LightCycler 480 system using the Brilliant II SYBR Green qRT-PCR kit (Agilent Technologies) according to the manufacturer’s instructions with HIV-1 *gag* SK462 (AGTTGGAGGACATCAAGCAGCCATGCAAAT) and SK431 (TGCTATGTCACTTCCCCTTGGTTCTCT). Relative HIV RNA copy numbers (viral load, VL) were normalized to levels of ribosomal S9 (*RPS9*) protein as determined by a separate qRT-PCR (forward: AAGGCCGCCCGGGAACTGCTGAC, reverse: ACCACCTGCTTGCGGACCCTGATA). Average relative gut VL was calculated as the mean VL across each measured gut compartment (transverse colon, terminal ileum, and duodenum). The limit of detection (10^−7^ relative copies) was used for compartments in which no HIV RNA was detected. Subjects were dichotomized into “undetectable” and “detectable” groups separately for PBMCs and for the gut. An individual with HIV viral load below the limit of detection in all measured gut compartments was placed in the “undetectable” group, otherwise they were categorized as “detectable”.

### Statistical analysis

Nonparametric tests were used to compare medians between groups. The Mann-Whitney test was used for comparisons of 2 groups and the Kruskal-Wallis test followed by Dunn’s post tests for >2 groups. Spearman rank order correlation coefficients were used to assess associations between continuous variables. Differences were considered significant at p < 0.05. Graphpad Prism 5 was used for all analyses except comparison of distributions of cytokine production, which was performed in SPICE using a partial permutation test for 10,000 iterations as described^46^.

## Electronic supplementary material

Supplementary Information

## Data Availability

All data generated or analyzed during this study are included in this published article (and its Supplementary Information files).
