# Supplementary material for: Increased frequency of systemic pro-inflammatory Vδ1+ γδ T cells in HIV elite controllers correlates with gut viral load
Source: Sci Rep. 2018 Nov 7;8:16471. doi: 10.1038/s41598-018-34576-4 (PMC6220338; doi:10.1038/s41598-018-34576-4)
Supplement: Supplementary file 1 — Supplementary Information [file 41598_2018_34576_MOESM1_ESM.pdf]

Supplementary Information

Increased frequency of systemic pro-inflammatory Vδ1$^+$ $\gamma\delta$ T cells in HIV elite controllers correlates with gut viral load

Gregory S. Olson[1], Sarah W. Moore[1], James M. Richter[2], John J. Garber[2], Brittany A. Bowman[1], Crystal A. Rawlings[1], Meaghan Flagg[1], Bjorn Corleis[1], Douglas S. Kwon[1]*

[1] The Ragon Institute of MGH, MIT and Harvard, Cambridge, Massachusetts, United States of America

[2] Division of Gastroenterology, Massachusetts General Hospital, Boston, Massachusetts, United States of America

*Correspondence to:

Dr. Douglas S. Kwon, Ragon Institute of MGH, MIT and Harvard, 400 Technology Square, Cambridge, MA, 02139, USA; dkwon@mgh.harvard.edu

Supplementary Table 1

	HIV Negative	Elite Controller	Chronic Untreated
**n**	16	13	11
**Age (years)**	43 ± 8	47 ± 8	40 ± 8
**Male (%)**	44	31	73
**CD4+ T cells (/µL)**		982 ± 511	558 ± 282
**Plasma VL (copies/mL)**			49,927 ± 85,713
**Days since diagnosis**		4,750 ± 1,906	4,910 ± 2,647
**HCV (#)**	0	5	2

Supplementary Table 1. Clinical characteristics of African American subjects. Values for age, CD4+ T cell count, and days since diagnosis represent mean ± standard deviation. HCV (#) represents the number of subjects co-infected with Hepatitis C virus. CD4+ T cell count and plasma VL not determined for HIV negative cohort.

Supplementary Table 2

Cohort	Age (years)	Gender	Race	CD4+ T cells (/µL)	Days since diagnosis	Plasma VL (cp/mL)	Average Gut VL (relative RNA)
**EC**	30	M	W	907	1600	ND	ND
**EC**	56	F	W	794	3202	ND	ND
**EC**	60	M	W	578	9910	53	ND
**EC**	54	M	W	1140	10001	ND	ND
**EC**	47	F	Other	778	8220	ND	4.00E-07
**EC**	44	M	W	1018	4863	164	7.33E-07
**EC**	64	M	W	712	10398	ND	5.67E-06
**EC**	57	M	W	1050	9101	ND	6.03E-06
**EC**	46	M	W	667	9755	24	6.57E-06
**EC**	41	M	>1	396	2547	ND	9.37E-06
**EC**	46	M	W	1167	4002	128	2.77E-05
**EC**	51	M	W	663	8976	ND	4.69E-03
**VC**	50	M	AA	789	4644	270	ND
**VC**	51	M	W	720	7083	135	ND
**HIV-**	33	M	>1				ND
**HIV-**	49	M	W				ND
**HIV-**	46	M	W				ND

Supplementary Table 2. Clinical characteristics of subjects with gut viral load data. EC, elite controller; VC, viremic controller; Neg, HIV uninfected; W, White; AA, African American; >1, more than one race. ND, viral RNA not detected; for relative RNA quantification, this was at a level of 1.00E-07. *One EC was co-infected with HCV.

Supplementary Figure 1

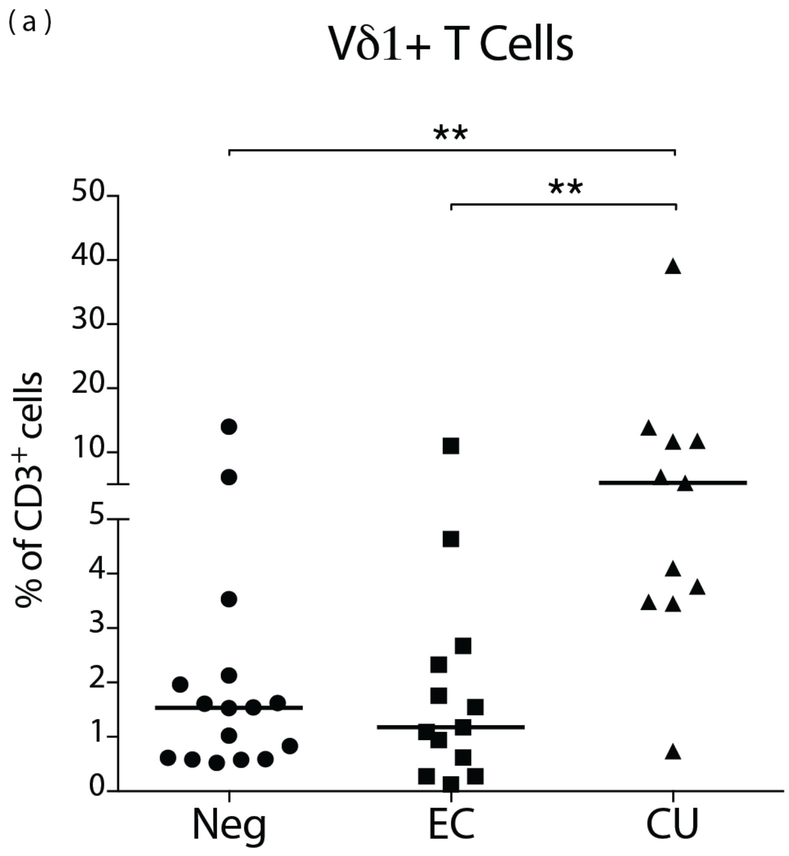

Supplementary Figure 1. African American Vδ1+ cells only expand with unsuppressed viremia. Thawed PBMCs were stained for flow cytometry and viable CD3+ cells were analyzed for expression of Vδ1. Summary data showing the median percentage of viable CD3+ cells that are Vδ1+ for HIV-uninfected (Neg) (n=16 subjects), EC (n=13), and chronic untreated (CU; n=11) subjects. The medians of the cohorts were significantly different (p=0.0027) as assessed by the Kruskal-Wallis test. Dunn's multiple comparison tests were used to assess differences between all groups. * p < 0.05; ** p <0.01; *** p < 0.0001.

Supplementary Figure 2

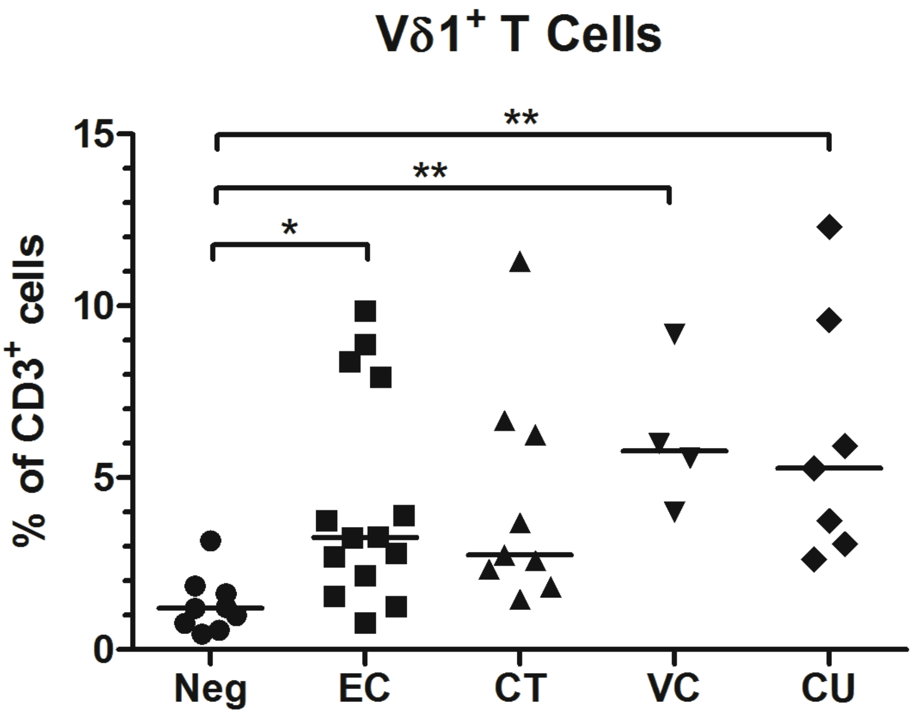

Supplementary Figure 2. Differences in gender composition between groups does not explain differences in Vδ1 frequency. Thawed PBMCs were stained for flow cytometry and viable CD3+ cells were analyzed for expression of Vδ1. Females were excluded from this analysis. Horizontal bars represent the median percentage in male cohorts of viable CD3+ cells that are Vδ1+ HIV-uninfected (Neg) (n=9 subjects), EC (n=14), CT (n=9), VC (n=4), and CU (n=7) subjects. The medians of the cohorts were significantly different (p=0.0015) as assessed by the Kruskal-Wallis test. Dunn's multiple comparison tests were used to assess differences between all groups. * $p < 0.05$; ** $p < 0.01$; *** $p < 0.0001$.

Supplementary Figure 3

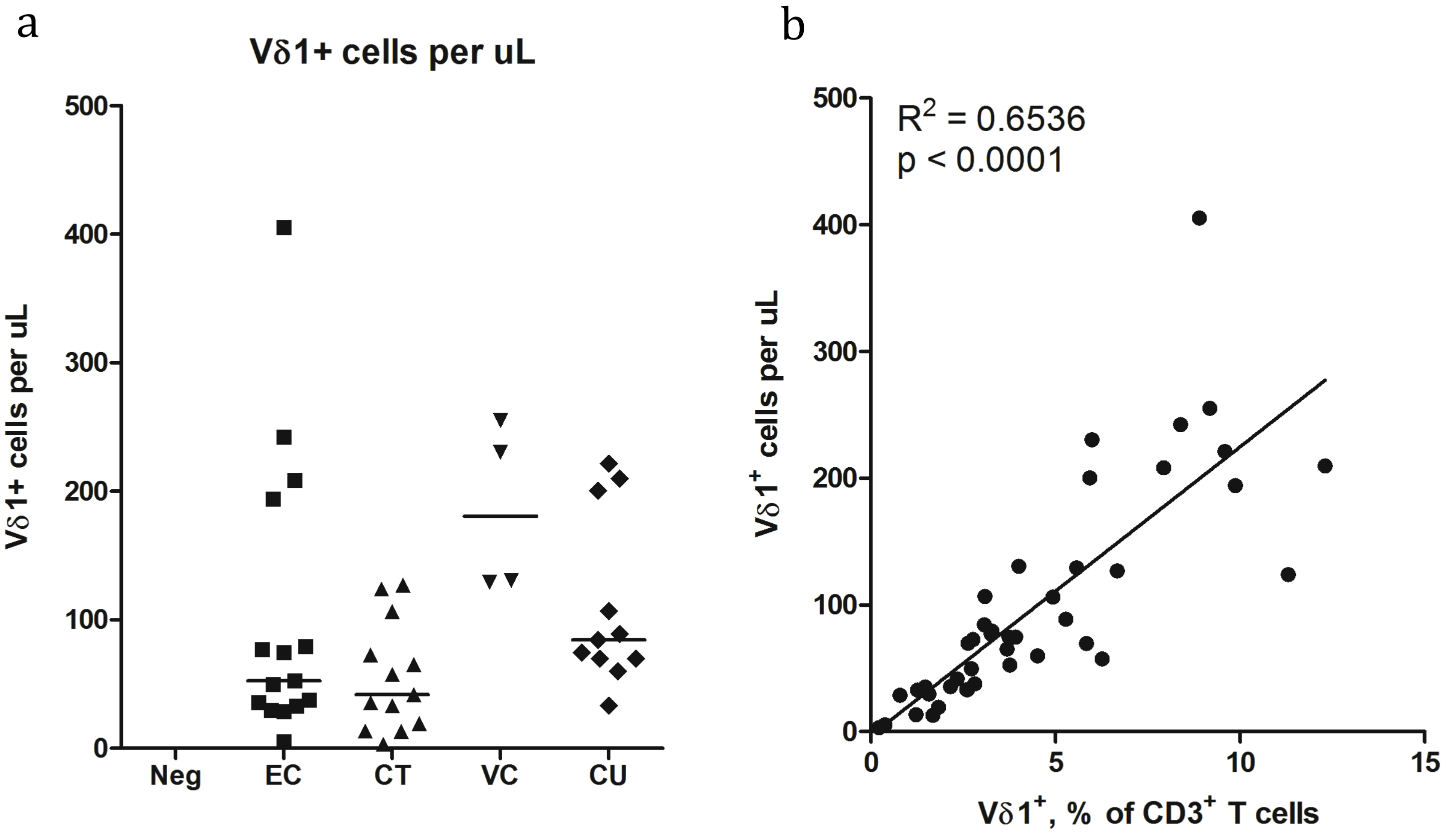

Supplementary Figure 3. Increases in Vδ1 frequency represent an expansion of absolute numbers of Vδ1 cells. Clinical CD4 cell counts in HIV infected individuals were used to approximate Vδ1 cell counts by the following formula: Vδ1 cells/uL = CD4 count/uL * (1/CD4+ frequency of CD3+ cells) * Vδ1 frequency of CD3+ cells. a) The absolute counts of the HIV infected cohorts were plotted for white subjects. The pattern of Vδ1 cell counts in these cohorts mirrored that of Vδ1 cell frequency (see Fig 1b). b) The calculated Vδ1 cell counts are plotted against the Vδ1 cell frequency of CD3+ cells, showing a significant Pearson correlation between these measurements of Vδ1 cell expansion.

Supplementary Figure 4

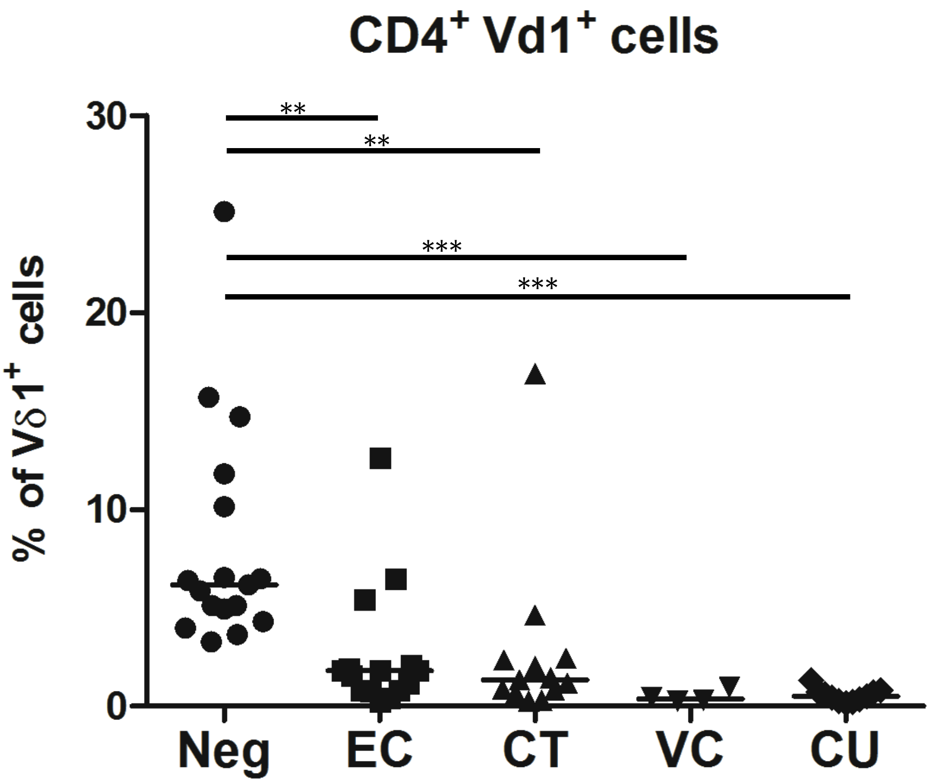

Supplementary Figure 4. A small proportion of Vδ1 cells express CD4. CD4 expression on Vδ1 cells was assessed by flow cytometry. The medians of the cohorts (horizontal lines) were significantly different ($p<0.0001$) as assessed by the Kruskal-Wallis test. Dunn's multiple comparison tests were used to assess differences between pairwise groups. ** $p <0.01$; *** $p < 0.0001$.

Supplementary Figure 5

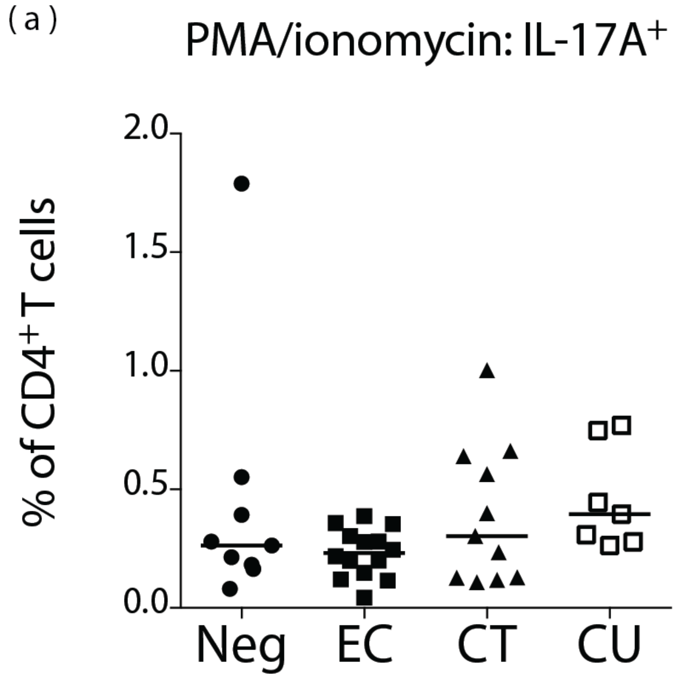

Supplementary Figure 5. Peripheral CD4+ T cells produce IL- 17A upon PMA/ionomycin stimulation. Thawed PBMCs were stimulated with PMA/ionomycin for 6 hours and cytokine production measured by intracellular cytokine staining. Summary data showing the median percentage of Vδ1-neg, Vδ2-neg, CD4+ T cells that produce IL-17A for HIV-uninfected (Neg) (n= 9 subjects), EC (n=14), chronic treated (CT; n=11), and chronic untreated (CU; n=7) subjects.

Supplementary Figure 6

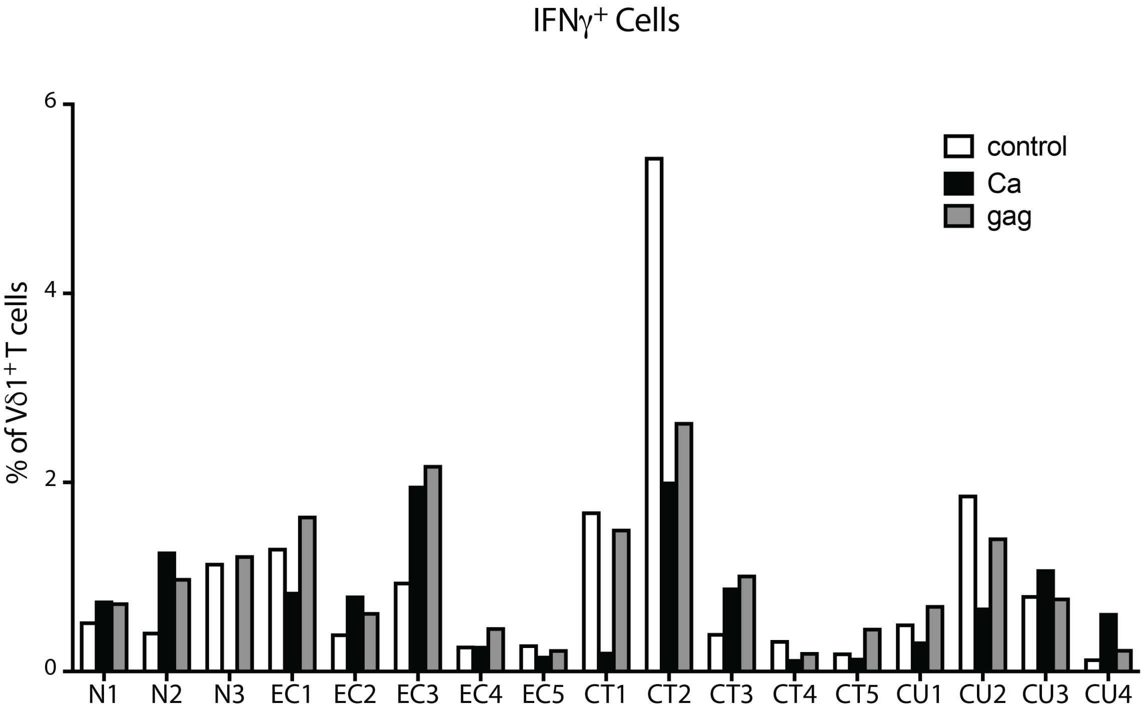

Supplementary Figure 6. IFNγ production in Vδ1+ cells in response to various stimuli. Thawed PBMCs were stimulated under various conditions (control: media alone, Ca: heat-killed Candida albicans. gag: peptide pools of HIV gag) for 6 hours and cytokine production measured by intracellular cytokine staining. Data showing the percentage of Vδ1+ T cells that produce IFNγ for representative HIV-uninfected (N), elite controller (EC), and chronic untreated (CU) subjects.

Supplementary Figure 7

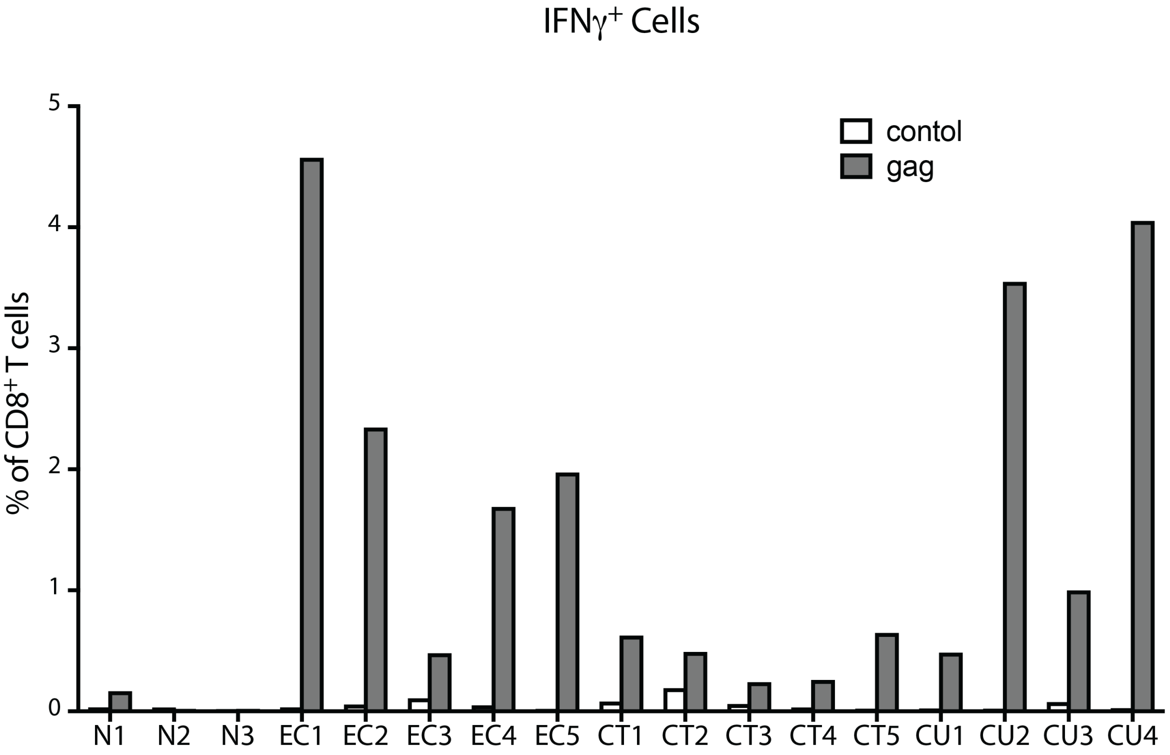

Supplementary Figure 7. IFNγ production in CD8+ T cells in response to stimulation. Thawed PBMCs were stimulated with either media alone (control) or gag peptide pools (gag) for 6 hours and cytokine production measured by intracellular cytokine staining. Data showing the percentage of Vδ1-neg and Vδ2-neg CD8+ T cells that produce IFNγ for representative HIV-uninfected (N), elite controller (EC), and chronic untreated (CU) subjects.

Supplementary Figure 8

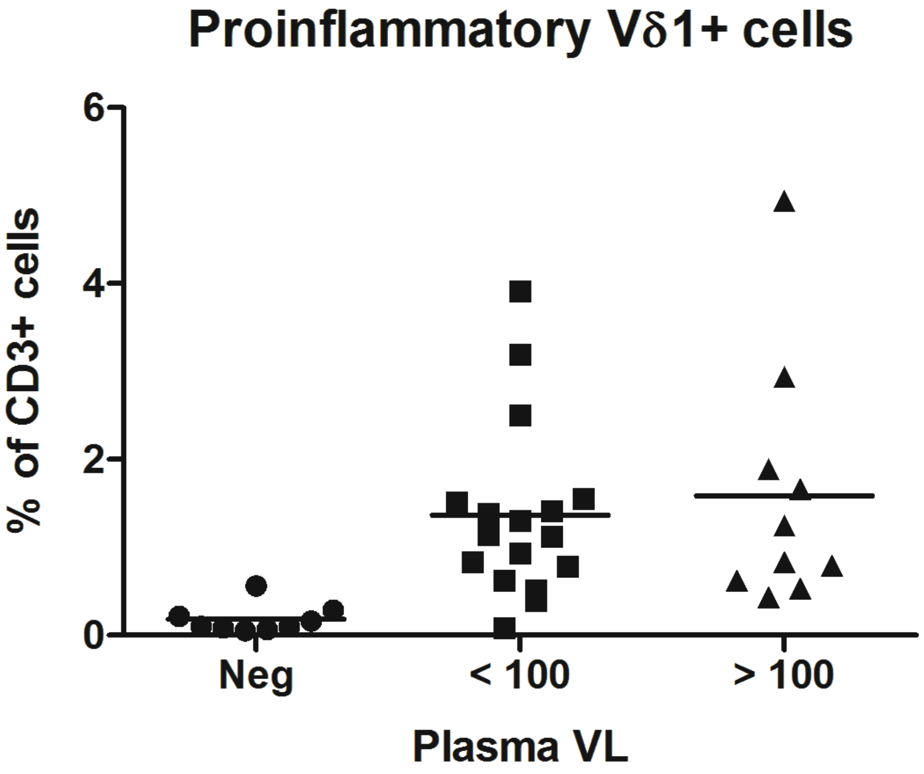

Supplementary Figure 8. Plasma VL does not explain increase in frequency of pro-inflammatory Vδ1^{+} cells. HIV-infected subjects were dichotomized based on the plasma viral load measured by the clinical lab into groups with low (<100 cp/mL) and high (>100 cp/mL) plasma viremia and the frequencies of cytokine-producing Vδ1 cells were compared to uninfected controls (Neg). While both HIV infected groups had significantly more pro-inflammatory Vδ1^{+} cells, there was no significant difference between the groups dichotomized by plasma viremia.

Supplementary Figure 9

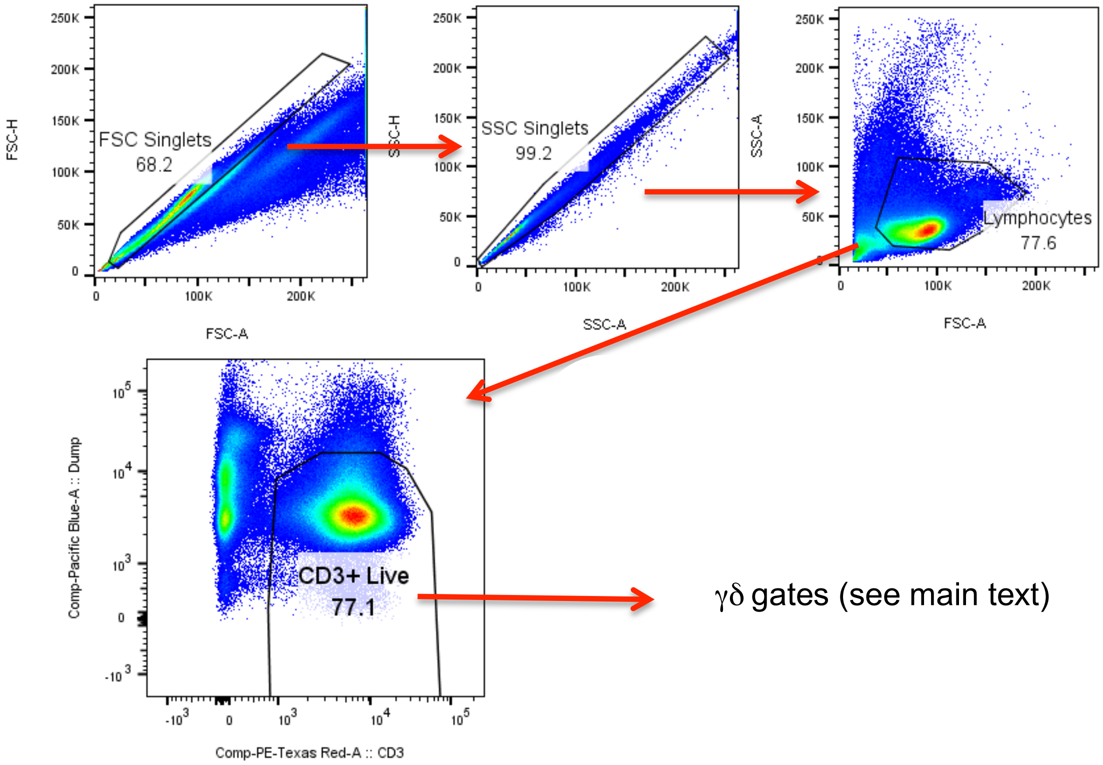

Supplementary Figure 9. Full gating scheme for identifying γδ cells.
